# Prediction of adolescent idiopathic scoliosis with machine learning algorithms using brain volumetric measurements

**DOI:** 10.1002/jsp2.1355

**Published:** 2024-07-15

**Authors:** Ahmet Payas, Hikmet Kocaman, Hasan Yıldırım, Sabri Batın

**Affiliations:** ^1^ Faculty of Medicine, Department of Anatomy Amasya University Amasya Turkey; ^2^ Faculty of Health Sciences, Department of Physiotherapy and Rehabilitation Karamanoglu Mehmetbey University Karaman Turkey; ^3^ Faculty of Kamil Özdağ Science, Department of Mathematics Karamanoğlu Mehmetbey University Karaman Turkey; ^4^ Orthopedics and Traumatology Department Kayseri City Education and Training Hospital Kayseri Turkey

**Keywords:** brain, diagnosis, machine learning, magnetic resonance imaging, scoliosis

## Abstract

**Background:**

It is known that neuroanatomical and neurofunctional changes observed in the brain, brainstem and cerebellum play a role in the etiology of adolescent idiopathic scoliosis (AIS). This study aimed to investigate whether volumetric measurements of brain regions can be used as predictive indicators for AIS through machine learning techniques.

**Methods:**

Patients with a severe degree of curvature in AIS (*n* = 32) and healthy individuals (*n* = 31) were enrolled in the study. Volumetric data from 169 brain regions, acquired from magnetic resonance imaging (MRI) of these individuals, were utilized as predictive factors. A comprehensive analysis was conducted using the twelve most prevalent machine learning algorithms, encompassing thorough parameter adjustments and cross‐validation processes. Furthermore, the findings related to variable significance are presented.

**Results:**

Among all the algorithms evaluated, the random forest algorithm produced the most favorable results in terms of various classification metrics, including accuracy (0.9083), AUC (0.993), f1‐score (0.970), and Brier score (0.1256). Additionally, the most critical variables were identified as the volumetric measurements of the right corticospinal tract, right corpus callosum body, right corpus callosum splenium, right cerebellum, and right pons, respectively.

**Conclusion:**

The outcomes of this study indicate that volumetric measurements of specific brain regions can serve as reliable indicators of AIS. In conclusion, the developed model and the significant variables discovered hold promise for predicting scoliosis development, particularly in high‐risk individuals.

## Graphical abstract

Adolescent idiopathic scoliosis does not cause much pain. This may cause scoliosis to progress insidiously and cause late diagnosis. Treatment of advanced scoliosis is both costly and difficult. In this study, we tried to show that scoliosis can be predicted by artificial intelligence using the brain volumes of individuals with scoliosis.

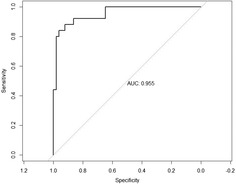

## 1 INTRODUCTION

Adolescent idiopathic scoliosis (AIS) is a three‐dimensional spinal deformity that typically involves one or more parts of the spine.^1^ Its etiology remains not fully understood, and it predominantly affects females aged between 10 and 18.^1^

AIS is categorized based on the size, location, and direction of the curvature, and if left untreated, it can lead to serious health problems such as back and waist pain, shortness of breath, bloating and fatigue.^2^ Timely diagnosis of AIS is crucial in preventing subsequent deformities that may develop over time.^3^ When AIS is identified in its early stages, non‐surgical treatments are viable. Moreover, early diagnosis and treatment of AIS with cost‐effective methods, such as exercise, alleviate the substantial financial burden on both individuals and society.^4^

Recent studies over the past two decades have increasingly explored the role of the nervous system in the development of AIS.^2^, ^5^ It is suggested that the connection between brain anomalies and spinal deformities is primarily attributed to neuroanatomical and neurofunctional alterations observed in the brain, brainstem, and cerebellum.^2^ Research has provided evidence of variations in both the cortex and white matter structure of the brain and cerebellum in individuals with AIS compared to their healthy counterparts.^6^, ^7^ Additionally, it has been noted that cortico‐cortical inhibition (It is the suppression of some unwanted impulses produced by the cerebral cortex by the cerebral cortex) is significantly reduced on the concave side of the curvature in individuals with AIS.^8^ These collective findings underscore the association between the central nervous system and AIS.^5^

Electroencephalography, MRI, and transcutaneous electrical stimulation methods have all been used to identify variations in the central nervous system in individuals with AIS.^9^ MRI is a non‐invasive imaging technology that creates detailed 3D anatomical images through the use of magnets and radio frequency waves.^10^ MRI serves to assess the human brain from both structural and functional perspectives, utilizing techniques such as volumetric analysis, shape analysis, voxel‐based morphometry calculations, cortical thickness measurement, tissue analysis, diffusion tensor imaging, and functional MRI.^11^

Machine learning (ML)‐based modeling is a relatively novel analytical method that has gained prominence, primarily in the development of predictive models within the domain of medical research.^10^, ^12^ ML is predominantly leveraged in medical research for disease classification, clinical decision‐making, and the formulation of novel treatment strategies.^13^, ^14^ Despite the increasing momentum in medical research using ML, the existing body of research on AIS is still notably lacking. In the context of AIS, several investigations have used ML models to predict the progression or severity of scoliosis.^15^, ^16^, ^17^ Wang et al.^15^ developed a deep learning model for predicting the progression of AIS during the initial clinic visit. Furthermore, another study used a ML approach to classify scoliosis patients based on their trunk surface asymmetry patterns.^16^ Additionally, a random forest (RF) model was formulated to identify critical prognostic features for curve progression and predict the final major Cobb angle.^17^ Furthermore, previous research has focused on assessing cortical thickness and brain white matter in individuals with AIS using brain MRI.^6^, ^7^ However, as of now, there has been a notable absence of ML‐based models for predicting AIS based on brain MRI volume measurements. Comprehensive investigation of brain volume measurements using ML algorithms in individuals with AIS may allow a clearer understanding of the role of the brain in the etiology of AIS. It may also contribute to understanding the link between brain region volumetric changes and the development of AIS in individuals. In this way, it can contribute significantly to the treatment management of individuals who may develop AIS. This entails the utilization of volume data derived from 169 distinct brain regions acquired from MRI images of individuals with mean age range of (15 ± 2.5) in the healthy group and age range of (15.6 ± 2.1) in the patient group. The details related to the characteristics of the demographic data are also presented in Table 1.

**TABLE 1:** Demographic characteristics of the participants.

	Group				*p*
	Healthy (*n* = 31)		Patient (*n* = 32)		
	Mean	SD	Mean	SD	
Age (Year)	15.9	2.5	15.6	2.1	0.561
BMI (kg/m^2^)	20.8	1.6	20.5	2.3	0.888
Mean angle of the major curve (Cobb^○^)	‐	‐	52.5	6.6	‐

Abbreviations: BMI, Body mass index; SD, Standard deviation.

The study aimed to estimate AIS using ML algorithms, as opposed to traditional statistical approaches. For this purpose, volume data obtained from 169 different brain regions of AIS and healthy individuals were processed in ML algorithms. It was aimed to predict the onset of AIS by comparing the data of AIS and healthy individuals.

## 2 MATERIALS AND METHODS

### 2.1 Study design

The cross‐sectional study was carried out on girls with severe curvature who were recommended scoliosis surgery by an orthopedist and healthy girls with similar demographic characteristics. Before starting the study, ethical approval was obtained from Hitit University Ethics Committee (2022–23, date: 04.11.2022). Since all participants were under the age of 18, written and verbal consent was obtained from the parents of the participants for their participation in the study. The study was conducted in accordance with the Helsinki Declaration.

### 2.2 Participants

A total of 63 individuals, 32 with AIS and 31 healthy individuals, who were diagnosed with AIS at the Orthopedics and Traumatology Polyclinic of Kayseri City Training and Research Hospital, were included in the study.

Since AIS is more common in female gender, only female participants were included in the study to minimize gender and hormonal differences. The inclusion criteria for individuals with AIS were as follows: being right‐handed dominant, having Lenke Type I scoliosis (the apex of the major curvature is on the right side), having a major curvature angle between 30–70 degrees, and having scoliosis diagnosed with AIS. Exclusion criteria in the study were determined as follows: Using the left hand as the dominant hand; presence of scoliosis other than AIS; the presence of neurological, psychiatric, muscular, rheumatic or orthopedic diseases.

### 2.3 Data acquisition

MRI imaging of the participants was performed with the Dutch brand 3 T (Tesla) Siemens Magnetom Skyra device. Participants' MRI and Diffusion tensor imaging (DTI), (It is used to obtain information about the microarchitecture of brain white matter tissue), sequence settings were as follows; TR = 4900 ms, TE = 95 ms, Number‐of‐Slice = 36, Flip Angle = 90o, FOV = 230 × 230 mm2, matrix = 128 × 128 and slice thickness = 3.0 mm (voxel size 1.8 × 1.8 × 3.5 mm). The resulting images were converted to DICOM format and saved.

### 2.4 Data processing

The parcel part of the study: it was performed in MriStudio and MriCloud software using MRI data in DICOM format. The segmentation process was started by converting the MRI data in DICOM format to “.dpf” format. After these procedures, the entry was made by selecting “DTI processing” under the diffusion tensor imaging (“DTI”) tab on the http://www.braingps.mricloud.org website. The files previously converted to “.dpf” format were converted to “.zip” format, uploaded to the relevant area and sent to the server. The file, which was processed by the server, was downloaded in “.zip” format with an ID number. The files named “DtiSeg_tensor_fa.hdr or DtiSeg_tensor_fa.img” in the “.zip” format file were opened in the ROIEditor program. Following these operations performed in ROIEditor, a parcelation map was opened on the images to be parceled, and measurements and calculations were made for 168 regions of the brain.

MriStudio (https://MriStudio.org) program: it consists of three separate software: DtiStudio for opening and saving images, ROIEditor for creating masks from images, and DiffeoMap for linear and non‐linear image transformation.

In the MriStudio program, it is important to correctly enter the participant's age and gender and the brand information of the MRI device on which the MRI is performed.

### 2.5 Experimental settings

Two stages were followed in the data analysis process of the study. The first stage covers statistical comparisons of the volume measurements of left and right cerebral hemispheres between the groups. The main objectives of this stage are to acquire an idea about the dispersion of the measurements, to observe possible group differences in a statistical perspective, and to derive baseline insights for machine learning models, which is the main focus of this study. Therefore, following the first stage, comprehensive performance comparisons of ML algorithms have been conducted in the second stage.

### 2.6 Statistical analysis

The statistical tests were conducted on IBM SPSS 25 and MedCalc 21 softwares. In the comparison of cerebral hemisphere measurements among the healthy and patient groups, independent samples *t* test, Welch *t* test or Mann–Whitney *U* test were performed depending on whether the assumptions of normality and homogeneity of variance were met, and accordingly, the appropriate test result was reported. The distribution of the data (i.e. normality), which is one of the parametric test assumptions, was tested by Kolmogrov–Smirnov test and homogeneity of variance was tested by Levene test. Statistical significance value was set at 0.05.

#### 2.6.1 Machine learning algorithms

In the second stage, eleven different ML algorithms logistic regression,^18^ naive bayes, k nearest neighbors,^19^ support vector machines,^20^ RF,^21^ linear discriminant analysis,^22^ multilayer perceptron,^23^ C5.0,^17^ bagging,^21^ extreme gradient boosting^24^ and MARS^25^ were evaluated. The data set was randomly shuffled and split into two thirds training data and the rest test data. In training the models, training data based on five times repeated 5‐fold cross validation was used and independent test data for testing. The data were preprocessed using standardization (i.e. centering and scaling) to eliminate the effect of unit variations. A grid search was used for the tuning parameters corresponding to each ML model and thirty possible combinations were tested. The model performance criteria are measured as Accuracy, AUC (area under the roc curve), F1 and Brier scores with confidence intervals. The variable importance plots and ROC curve are finally presented utilizing the best model. During the determination of the best model, ROC curve is a quite beneficial and commonly used criterion in terms of assessing the performance of a classifier by illustrating the trade‐off between the true positive rate and the false positive rate across various threshold settings. The results of the analysis were obtained using R software^26^ as well as tidyverse^27^ and tidymodels^28^ packages.

## 3 RESULTS

Demographic characteristics such as age and body mass index were similar in both groups (*p* > 0.05) (Table 1).

The statistical analysis between the healthy and patient groups revealed that *cuneus, cerebellum, corticospinal tract, inferior fronto‐occipital fasciculus, external capsule, body of corpus callosum, splenium of corpus callosum, retrolenticular part of internal capsule and pons* measurements of the right cerebral hemisphere were statistically significant (p < 0.05) (Appendix A, Table S4). The statistical analysis between the healthy and patient groups revealed that *parahippocampal gyrus, splenium of corpus callosum, nucleus accumbens and tapatum* measurements of the left cerebral hemisphere were statistically significant (*p* < 0.05) (Appendix A, Table S5).

**FIGURE 1:**
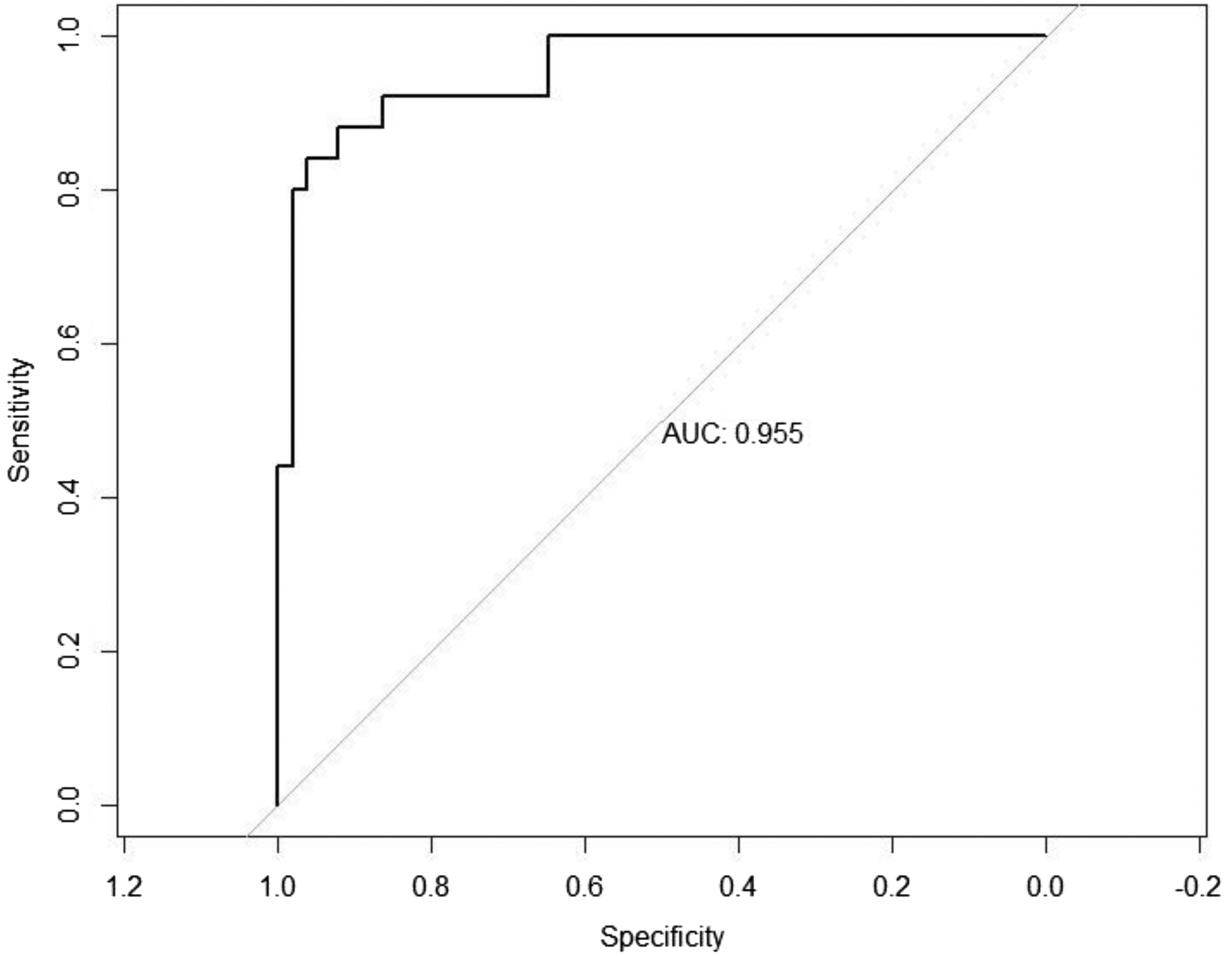
The ROC curve for the RF model.

Performance comparisons of ML algorithms for both training and test data based on right cerebral hemisphere measurements are given in Tables 2 and 3 respectively. From the criteria evaluated in the study, it is seen that the RF algorithm is the best algorithm in both training and test data when the Accuracy, AUC and F1‐score values are high and the Brier score is low as a measure of a good model. The training performance of the RF algorithm is quite convincing with an accuracy of 0.9083 (CI: 0.8679, 0.9488), AUC of 0.993 (CI: 0.9870, 0.9990), F1‐score of 0.9170 (CI: 0.8794, 0.9546) and Brier score of 0.1256 (CI: 0.1151, 0.1360). Similarly, it is seen that it provides considerably high results (Accuracy: 0.8500, AUC: 0.9550, F1‐score: 0.9612 and Brier score: 0.1622) in the test data. In addition, the ROC curve obtained using the test data is given in Figure 1 to support that the performance is reasonable and good.

**TABLE 2:** Performance metrics (mean and 95% confidence interval) of algorithms on repeated cross‐validation of training set for right cerebral hemisphere measurements.

Model/metric	Accuracy	AUC	Brier	F1‐score
Logistic Regression	0.6783 (0.6033, 0.7533)	0.730 (0.6439, 0.8161)	0.3176 (0.2428, 0.3924	0.6944 (0.6169, 0.7720)
Naive Bayes	0.7300 (0.6662, 0.7938)	0.835 (0.7717, 0.8983)	0.2305 (0.1726, 0.2884	0.7567 (0.6918, 0.8217)
KNN	0.5350 (0.4543, 0.6157)	0.645 (0.5526, 0.7374)	0.2681 (0.2222, 0.3140	0.5355 (0.4389, 0.6320)
Svm (Polynomial)	0.8717 (0.8272, 0.9161)	0.950 (0.9122, 0.9878)	0.0943 (0.0690, 0.1197	0.8891 (0.8490, 0.9292)
Svm (Radial)	0.8033 (0.7393, 0.8674)	0.910 (0.8515, 0.9685)	0.1493 (0.1191, 0.1795	0.8128 (0.7410, 0.8846)
Random Forest	**0.9083 (0.8679, 0.9488)**	**0.9930 (0.9870, 0.9990)**	**0.1256 (0.1151, 0.1360)**	**0.9170 (0.8794, 0.9546)**
LDA	0.7200 (0.6549, 0.7851)	0.820 (0.7444, 0.8956)	0.2034 (0.1541, 0.2528	0.7197 (0.6562, 0.7833)
MLP	0.8517 (0.8033, 0.9001)	0.895 (0.8508, 0.9392)	0.1409 (0.1194, 0.1624	0.8612 (0.8151, 0.9073)
C5	0.8333 (0.7829, 0.8837)	0.830 (0.7776, 0.8824)	0.1667 (0.1163, 0.2171	0.8582 (0.8115, 0.9048)
Bagging	0.8550 (0.8060, 0.9040)	0.920 (0.8750, 0.9650)	0.1115 (0.0869, 0.1360	0.8762 (0.8326, 0.9198)
XGBoost	0.9083 (0.8654, 0.9513)	0.980 (0.9660, 0.9940)	0.0643 (0.0401, 0.0886	0.9204 (0.8816, 0.9592)
MARS	0.7650 (0.7060, 0.8240)	0.865 (0.8020, 0.9280)	0.2204 (0.1657, 0.2750	0.7807 (0.7264, 0.8350)

Abbreviation: AUC, area under the receiver operating curve. “Bold” indicates the best performing model.

**TABLE 3:** Performance metrics (mean and 95% confidence interval) of algorithms performance based on independent testing set for right cerebral hemisphere measurements.

Model/metric	Accuracy	AUC	Brier	F1‐score
Logistic Regression	0.4200 (0.3237, 0.5163)	0.3889 (0.2793, 0.4985)	0.5737 (0.4789, 0.6685)	0.4815 (0.3449, 0.6181)
Naive Bayes	0.5400 (0.4376, 0.6424)	0.6889 (0.5493, 0.8285)	0.4152 (0.3194, 0.5110)	0.6970 (0.5693, 0.8246)
KNN	0.3600 (0.2651, 0.4549)	0.5667 (0.4190. 0.7144)	0.3193 (0.2621, 0.3765)	0.4086 (0.2581, 0.5591)
Svm (Polynomial)	0.8200 (0.7307, 0.9093)	0.9456 (0.9284, 0.9627)	0.1920 (0.1742, 0.2098)	0.9185 (0.8618, 0.9752)
Svm (Radial)	0.6200 (0.5229, 0.7171)	0.8444 (0.7352, 0.9537)	0.2291 (0.1875, 0.2708)	0.7803 (0.7086, 0.8520)
Random Forest	**0.8500 (0.7679, 0.9321)**	**0.9550 (0.9402, 0.9712)**	**0.1622 (0.1101, 0.2142)**	**0.9612 (0.9283, 0.9942)**
LDA	0.7000 (0.5970. 0.8030)	0.8000 (0.6794, 0.9206)	0.2051 (0.1676, 0.2425)	0.8291 (0.7285, 0.9297)
MLP	0.7500 (0.6542, 0.8458)	0.9333 (0.8781, 0.9885)	0.1763 (0.1355, 0.2170)	0.9189 (0.8535, 0.9843)
C5	0.6600 (0.5553, 0.7647)	0.6889 (0.5869, 0.7908)	0.3400 (0.2353, 0.4447)	0.7928 (0.6792, 0.9064)
Bagging	0.6900 (0.5874, 0.7926)	0.8000 (0.6881, 0.9119)	0.2075 (0.1546, 0.2604)	0.8167 (0.7184, 0.9149)
XGBoost	0.7300 (0.6343, 0.8257)	0.8556 (0.7678, 0.9433)	0.1843 (0.1295, 0.2390)	0.8718 (0.7920. 0.9516)
MARS	0.5600 (0.4470. 0.6730)	0.6444 (0.5229, 0.7660)	0.4339 (0.3244, 0.5433)	0.6930 (0.5663, 0.8197)

Abbreviation: AUC, area under the receiver operating curve. “Bold” indicates the best performing model.

**FIGURE 2:**
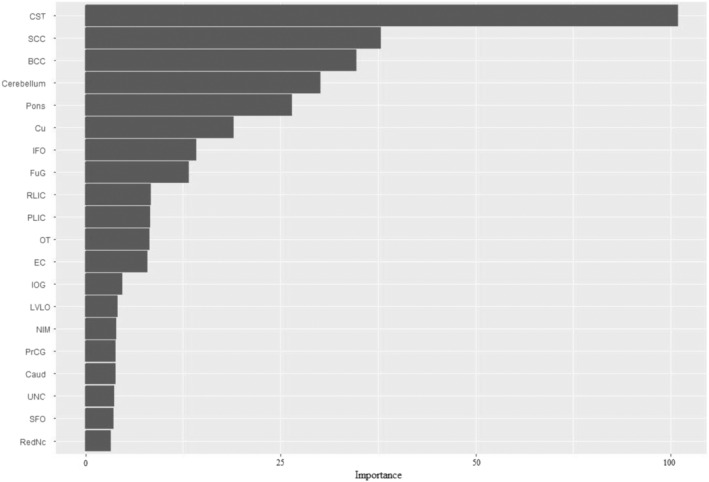
Relative importance scores of the twenty most important variables based on the RF model of the right cerebral hemisphere.

By using the RF algorithm's internal variable importance scores, the variable importance plot of the most important factors on whether an individual has scoliosis or is healthy is given in Figure 2. This plot displays the twenty most important factors. The first five of these factors are *corticospinal tract*, *body of corpus callosum, splenium of corpus callosum*, *cerebellum and pons* measurements, respectively. It is notable that the variables considered important by the RF algorithm are also statistically significant variables. Unlike statistical significance tests, measurements such as right fusıform gyrus, posterior limb of internal capsule, right optic tract were found to be significant in the ML model.

Statistical analysis and ML results based on left cerebral hemisphere measurements were separately obtained to provide additional insights. It is observed that *parahippocampal gyrus, splenium of corpus callosum, tapatum, and nucleus accumbens* measurements are statistically significant in the left cerebral hemisphere measurements. In general, there are relatively few significant results in the left cerebral hemisphere compared with the right hemisphere (Appendix B, Table S5). The results of ML algorithms are analyzed and it is found that they produce very weak and practically non‐functional values. The values achieved on the training dataset (Appendix B, Table S5) were relatively reasonable, but did not yield generalizable results for all of the criteria considered on the test dataset (Appendix C, Table S6). Results show that the highest accuracy and F1‐score are obtained in logistic regression (accuracy: 0.6300, F1‐score: 0.7197), the highest AUC is obtained in linear discriminant analysis (AUC: 0.7556) and the lowest Brier score is obtained in RF (Brier: 0.2378). The results of left cerebral hemisphere measurements are therefore omitted from the main focus of the study due to the low performance values and the fact that no single algorithm appears to be dominant (Table S7).

## 4 DISCUSSION

In the current study, ML‐based modeling approaches were used to predict scoliosis based on volumetric brain features. The results indicated that the RF model showed the best performance. Moreover, the most important variables were found to be *right corticospinal tract, right body of corpus right callosum, right splenium of corpus callosum, right cerebellum*, and *right pons* volumetric measurements, respectively (Figure 2).

ML algorithms have emerged as a promising tool for improving the prediction and early diagnosis of scoliosis. Several studies have explored the development and implementation of algorithms based on deep learning and ML in the context of scoliosis. These investigations have predominantly used data derived from spine radiographs for the purpose of assessing curve progression and predicting disease exacerbation in individual patients.^15^, ^17^, ^24^ In the current study, different from previous studies, we first used volumetric measurements of brain areas to test whether ML algorithms can distinguish individuals with scoliosis from healthy individuals and to test the performance of volumetric measurements of brain regions in predicting scoliosis. Twelve distinct ML models, including logistic regression, naive Bayes, RF, and XGBoost, were utilized. Nevertheless, in the current study, more favorable results in scoliosis prediction were achieved through the application of the RF model (Tables 2 and 3).

While the precise etiopathogenesis of AIS remains elusive, the disparities observed within the central nervous system underline the undeniable role of the brain in the development of scoliosis. In the study, it was observed that individuals with Lenke Type I AIS had differences in the volumes of 9 regions in the right hemisphere (Table S4) and four regions in the left hemisphere of the brain (Table S5) compared to healthy individuals. These differences in brain regions of individuals with AIS underline the undeniable role of the brain in the etiopathogenesis of AIS.

Significantly, the most pronounced variations, relative to healthy individuals, were observed within the right hemisphere, particularly in the regions governing the concave side of the curvature. Notably, substantial distinctions emerged in the neural pathways responsible for intra‐hemispheric and inter‐hemispheric connectivity, particularly within the areas associated with vision and motor control. It has been postulated that issues related to postural balance contribute to the etiopathogenesis and progression of scoliosis.^29^

In the present study, distinct variations were observed in several brain regions associated with vision, further underlining the potential link between AIS and visual perception. Notably, the cuneus (Brodmann area 17) in the *occipital lobe*, a region crucial for primary visual processing, showed differences. Similarly, the *fusiform gyrus*, responsible for advanced visual information processing, particularly in face recognition, exhibited variances. Additionally, differences were detected in the *retrolenticular part of the internal capsule*, connected to the optic radiation and vision, as well as the volume of the *right optic tract*, the pathway responsible for carrying sensory information. The *inferior occipital gyrus*, associated with visual processing, also displayed distinctions when compared to healthy individuals (Tables S4 and S5).

Achieving postural balance necessitates the effective transmission of sensory inputs from the visual, vestibular, and somatosensory systems to the brain.^30^ The sense of vision serves as an essential information source, conveying stimuli from both within the body and the external environment.^31^ In the case of visually impaired individuals, disruptions in normal head position and shoulder symmetry have been reported, often leading to conditions such as genu valgus in the knees and the development of spinal deformities like scoliosis, thoracic kyphosis, or lumbar lordosis.^32^ Additionally, Catanzariti et al.^33^ reported a fivefold increase in scoliosis cases among visually impaired individuals compared to the control group. Batin et al.^34^ documented a higher degree of visual field deviation in individuals with AIS than in the control group. Their study data also revealed asymmetry in right and left visual field tests among individuals with AIS.^34^

The study findings suggest that brain regions linked to visual processing could be a key source of vision‐related issues in individuals with AIS. The observed distinctions in brain regions responsible for the transmission and interpretation of visual sensations significantly strengthen the association between AIS and visual perception. Furthermore, the study highlights that the right hemisphere of individuals with AIS is more affected in terms of visual fields, which may account for the asymmetry observed in right and left visual field tests among these individuals.

In this study, more disparities were identified in the right brain hemisphere, which is responsible for the concave side of the curvature in individuals with AIS, in comparison to healthy individuals. Of particular note was the reduction in the volumes of the *gyrus precentralis* and *tractus corticospinalis*, both of which play critical roles in motor control (Tables S4 and S5).

The corticospinal tract, often referred to as the pyramidal tract, serves as the principal neural pathway facilitating voluntary motor function.^35^ Studies have reported that individuals with scoliosis exhibit asymmetry between the right and left corticospinal tracts. Additionally, it has been noted that both corticospinal tracts in individuals with scoliosis are weaker compared to their healthy counterparts.^5^, ^6^ In their study, Payas et al.^5^ associated the reduced number of fibers in the corticospinal tracts of individuals with scoliosis with decreased muscle strength. Furthermore, Kocaman et al.^36^ reported that muscle fibers on the convex side of the major curve in individuals with scoliosis are larger compared to the concave side, but the paraspinal muscle fibers on both sides of individuals with AIS are less developed compared to healthy individuals.

This decrease in gyrus precentralis and tractus corticospinalis volumes detected in this study may be closely related to the weaker muscles on the concave side of the curvature in individuals with scoliosis.

In this study, it was observed that individuals with AIS exhibited lower volumes in the right brain hemisphere, particularly in the *gyrus precentralis* and *tractus corticospinalis*, which are responsible for motor control on the concave side of the curvature. Conversely, the volumes of the *right red nucleus* and *right caudate nucleus*, which indirectly contribute to motor control, were found to be larger (Table S4).

In response to issues arising in any particular region, the brain often uses a compensation mechanism by adapting brain regions with similar functions to compensate for the affected area.^37^ The precentral cortex, located in the frontal lobe, is responsible for the voluntary control of motor movements and serves as the origin for numerous motor pathways, including the corticospinal tract, corticobulbar tract, and cortico‐rubrospinal tract.^38^ The caudate nucleus is a paired, “C”‐shaped subcortical structure located deep in the brain, near the thalamus. When combined with the putamen, the pair is referred to as the striatum, and they typically function collectively. The striatum is the primary source of input for the basal ganglia, which also encompasses the globus pallidus, subthalamic nucleus, and substantia nigra. Together, these deep brain structures predominantly regulate voluntary skeletal movement. Input to the caudate nucleus typically originates from the cortex, most frequently from the ipsilateral frontal lobe. Efferent projections from the caudate nucleus extend to the hippocampus, globus pallidus, and thalamus.^39^ Regarding the red nucleus, it primarily controls vertebrates lacking a significant corticospinal tract. However, in primates where the corticospinal system is dominant, it is considered vestigial.^40^ The human red nucleus plays a crucial role in various aspects of motor control.^41^

In this study, the increased volume in the *right red nucleus* and the *right caudate nucleus* may be indicative of the brain activating a compensation mechanism to compensate for the decrease in the volume of the *gyrus precentralis*. Consequently, it is reasonable to assume that motor dysfunction arising from the *gyrus precentralis* is compensated by the *right red nucleus* and the *right caudate nucleus*.

In the current study, it was observed that individuals with AIS exhibited lower volumes in several brain regions responsible for interhemispheric coordination, including the *splenium of the corpus callosum*, *body of the corpus callosum, inferior fronto‐occipital fasciculus*, and *superior fronto‐occipital fasciculus* (the latter could be a part of the *anterior internal capsule*), (Tables S4 and S5).

White matter primarily consists of axons enveloped by a lipid‐rich myelin sheath, which restricts diffusion and plays a crucial role in providing fast and efficient connections between the cortex and subcortical regions.^8^ Payas et al.,^5^ in their study, reported that individuals with scoliosis experienced notable issues, particularly in the white matter of the brain. There have been observations that the volume and the number of fibers in the corpus callosum, responsible for interhemispheric communication, are significantly reduced in individuals with AIS in comparison to their healthy counterparts.^6^ In a study by Joly et al., which involved individuals with AIS exhibiting right thoracic and right thoracic‐left lumbar curvature, it was reported that the anatomical location of the corpus callosum was lower than in healthy individuals.^42^ These findings support the notion that abnormalities in the brain's white matter play a role in the etiopathogenesis of AIS, leading to interhemispheric coordination deficits.

The study findings suggest that individuals with scoliosis may experience challenges in healthy communication between the right and left brain hemispheres, as well as within the same hemisphere. The observed issues in these brain structures might lead to compromised coordination between sensorimotor regions and hinder the ideal motor responses required for postural control.

### 4.1 Study limitations

The limitation of this study is that it included only female individuals with AIS. Additionally, the sample size of this study for individuals with AIS was relatively small, and the degree of curvature among the participants was notably severe, with an average curvature degree of 52.5. As a result, the findings should not be extrapolated to patients with milder curvatures. To address this limitation, further research is essential, involving a more extensive patient cohort that encompasses varying curvature severities and includes both genders. This will facilitate a more comprehensive understanding of AIS across a broader spectrum of cases.

## 5 CONCLUSION

This study has illustrated the robust predictive capabilities of the RF model in scoliosis prediction, particularly when considering the volumetric attributes of brain regions. Furthermore, it was discerned that the most influential variables for prediction were derived from the volumetric measurements of specific brain regions, including the right corticospinal tract, right corpus callosum body, right corpus callosum splenium, right cerebellum, and right pons. Additionally, the study identified that individuals with AIS exhibited more pronounced disparities in the right brain hemisphere associated with the concave side of the curvature. These findings suggest that, despite the primary impact on brain regions related to the sense of vision in individuals with scoliosis, motor functions and the white matter structures involved in intra‐hemispheric and inter‐hemispheric communication are also notably affected. It is important to note that delayed detection of scoliosis can lead to enduring damage to anatomical structures such as muscles, bones, joint capsules, and ligaments surrounding the spine. Given that AIS typically advances without causing pain and progresses rapidly, early diagnosis can be challenging. In light of the results presented in this study, the identification of regional disparities through brain MRI using ML algorithms holds promise for aiding in the diagnosis of AIS. This method may offer the potential to predict the development of scoliosis, particularly in individuals at higher risk.

## AUTHOR CONTRIBUTIONS

**Ahmet Payas**, PhD (Contribution: writing – original draft, visualization, methodology, investigation, formal analysis, data curation). **Hikmet Kocaman**, PhD (Contribution: Study design, writing – review & editing, formal analysis, data curation). **Hasan Yildirim**, PhD (Contribution: Writing – original draft, formal analysis, performed measurements, manuscript preparation). **Sabri Batin**, MD (Contribution: Evaluation of patients, Validation, supervision, methodology).

## FUNDING INFORMATION

This study was not funded by any commissions of public, commercial, or not‐for‐profit sectors for the conduct of research, study design, and collection, analysis, and interpretation of the data writing the report, and/or decision of the article for publication.

## CONFLICT OF INTEREST STATEMENT

The authors declare no conflicts of interest.

## INFORMED CONSENT

Written informed consent was obtained from each patient. The study was conducted in accordance with the principles of the Declaration of Helsinki.

## Supporting information


**Data S1:** Supporting Information.
