# Supplementary material for: Prediction of adolescent idiopathic scoliosis with machine learning algorithms using brain volumetric measurements
Source: JOR Spine. 2024 Jul 15;7(3):e1355. doi: 10.1002/jsp2.1355 (PMC11247394; doi:10.1002/jsp2.1355)
Supplement: Supplementary file 1 — Data S1: Supporting Information. [file JSP2-7-e1355-s001.docx]

**Supplementary Material (Appendices)**

**Appendix A**

| **Table 4.** Baseline characteristics of right cerebral hemisphere measurements | | | | | |
| --- | --- | --- | --- | --- | --- |
|  | **Group** | | | | **p** |
|  | **Healthy (n=31)** | | **Patient (n=32)** | |  |
|  | **Mean** | **SD** | **Mean** | **SD** |  |
| Superior parietal gyrus | 23904.6 | 2586.3 | 23473.1 | 2431.5 | 0.519 |
| Cingulate gyrus | 32566.6 | 2139.8 | 32605.6 | 2179.4 | 0.946 |
| Superior frontal gyrus | 57158.9 | 4496.6 | 56945.6 | 3278.4 | 0.838 |
| Middle frontal gyrus | 51649.5 | 3950.6 | 51244.1 | 4057.3 | 0.704 |
| İnferior frontal gyrus | 27353 | 2448.1 | 27523.1 | 2161.5 | 0.782 |
| Precentral gyrus | 31715.7 | 2936.4 | 31812.3 | 2324.6 | 0.890 |
| Postcentral gyrus | 26396.4 | 1663.4 | 26682.5 | 1932 | 0.554 |
| Angular gyrus | 22791 | 2043.1 | 22301 | 2030.7 | 0.368 |
| Pre-cuneus | 15418.4 | 1010 | 15742.5 | 1119.8 | 0.258 |
| Cuneus | 13042.2 | 1150 | 11753.2 | 1226.1 | **< .001** |
| Lingual gyrus | 16863.7 | 2050.4 | 17603.9 | 2530.4 | 0.233 |
| Fusiform gyrus | 29217.7 | 1696.2 | 29447.7 | 1986 | 0.642 |
| Parahippocampal gyrus | 1420,4 | 279.6 | 1328.9 | 216.1 | 0.170 |
| Superior occipital gyrus | 8327.3 | 1007.6 | 8233.6 | 1386.4 | 0.774 |
| Inferior occipital gyrus | 10490 | 1507.6 | 10203.6 | 1075 | 0.409 |
| Middle occipital gyrus | 29117.9 | 3151.5 | 29652 | 3468.3 | 0.547 |
| Entorhinal area | 1021.4 | 269 | 1017.2 | 277.3 | 0.954 |
| Superior temporal gyrus | 39600.7 | 2379 | 38833.9 | 2566.4 | 0.249 |
| Inferior temporal gyrus | 23733.2 | 1698.6 | 23670.2 | 1517.6 | 0.883 |
| Middle temporal gyrus | 45378.3 | 3275.9 | 45278 | 3052.6 | 0.905 |
| Lateral fronto-orbital gyrus | 11836 | 1176.6 | 11886 | 1097.9 | 0.869 |
| Middle fronto-orbital gyrus | 9198.1 | 860.1 | 9457.3 | 870.1 | 0.264 |
| Supramarginal gyrus | 23229.7 | 2109.2 | 23060.6 | 1626 | 0.734 |
| Gyrus rectus | 11368.1 | 975.9 | 11411.8 | 1253.8 | 0.885 |
| Insular | 8988.4 | 736.9 | 8959.3 | 605.6 | 0.871 |
| Amygdala | 2382.3 | 326.9 | 2454.9 | 390.2 | 0.452 |
| Hippocampus | 5171.7 | 520.5 | 5197.5 | 532.2 | 0.854 |
| Cerebellum | 97234.9 | 4165.1 | 89602.4 | 5879.6 | **< .001** |
| Corticospinal tract | 1350.7 | 209.2 | 920.7 | 162.5 | **< .001** |
| Inferior cerebellar peduncle | 1415.3 | 236.3 | 1385.3 | 220.1 | 0.622 |
| Medial lemniscus | 1678 | 281.6 | 1593.4 | 415.2 | 0.377 |
| Superior cerebellar peduncle | 1786.4 | 224.5 | 1806 | 209.7 | 0.735 |
| Cerebral peduncle | 2171 | 296.6 | 2189.9 | 377 | 0.835 |
| Anterior limb of internal capsule | 2575.8 | 370.6 | 2623.9 | 309.3 | 0.596 |
| Posterior limb of internal capsule | 5152.5 | 665.8 | 5345.4 | 568.8 | 0.243 |
| Posterior thalamic radiation | 12805.8 | 1385.5 | 13169.8 | 1242.4 | 0.300 |
| Anterior corona radiata | 9637.7 | 1896.1 | 9727.7 | 2116.5 | 0.867 |
| Superior corona radiata | 19487.6 | 1743.5 | 19321.1 | 1722 | 0.718 |
| Posterior corona radiata | 6274.5 | 888.6 | 6377.5 | 717.8 | 0.631 |
| Cingulum (cingulate gyrus) | 4013.2 | 645.8 | 3737.5 | 719 | 0.135 |
| Cingulum (hippocampus) | 2063 | 262.3 | 2070.2 | 272.2 | 0.920 |
| Fornix (cres) / Stria terminalis | 916.4 | 145.2 | 888.5 | 136.1 | 0.457 |
| Superior longitudinal fasciculus | 10202.6 | 1319.8 | 10063.7 | 1341.2 | 0.696 |
| Superior fronto-occipital fasciculus | 91.3 | 73.4 | 67.5 | 57.6 | 0.177 |
| Inferior fronto-occipital fasciculus | 2966.8 | 384.8 | 2407.9 | 507.7 | **< .001** |
| Sagittal stratum | 7222.7 | 958.5 | 7452.9 | 1032.7 | 0.388 |
| External capsule | 3276.4 | 408 | 2809.6 | 410.2 | **< .001** |
| Uncinate fasciculus | 1673.9 | 217 | 1776.6 | 240.9 | 0.098 |
| Pontine crossing tract | 1326 | 473.6 | 1440.7 | 411.6 | 0.332 |
| Middle cerebellar peduncle | 8966.1 | 1123 | 9339.1 | 1187.9 | 0.230 |
| Fornix (column and body of fornix) | 306.3 | 109.4 | 325.3 | 110.3 | 0.518 |
| Genu of corpus callosum | 3964.6 | 654.1 | 3821.9 | 495.7 | 0.355 |
| Body of corpus callosum | 4450 | 506.6 | 3684.7 | 422.7 | **< .001** |
| Splenium of corpus callosum | 8585.5 | 954.9 | 7170.4 | 896.6 | **< .001** |
| Retrolenticular part of internal capsule | 3052.6 | 334.6 | 2666.4 | 333.2 | **< .001** |
| Red nucleus | 59.4 | 34.9 | 64.8 | 25.9 | 0.505 |
| Substancia nigra | 251.4 | 101.9 | 275.5 | 74.4 | 0.309 |
| Tapatum | 1323.3 | 234.9 | 1259.7 | 258.6 | 0.338 |
| Caudate nucleus | 4991.7 | 605.1 | 5188.6 | 733.7 | 0.277 |
| Putamen | 4443.8 | 550.7 | 4319.5 | 346.4 | 0.307 |
| Thalamus | 9304.6 | 540.4 | 9324.5 | 684.5 | 0.904 |
| Globus pallidus | 983.3 | 132.7 | 999.7 | 126 | 0.635 |
| Midbrain | 2741.3 | 300.3 | 2769 | 288 | 0.723 |
| Pons | 994.3 | 170.7 | 729.5 | 160 | **< .001** |
| Medulla | 3198.2 | 392.8 | 3200.1 | 387.8 | 0.985 |
| Anterior commissure | 479.4 | 124.8 | 476.6 | 107.1 | 0.927 |
| Ansa lenticularis | 1165.8 | 194.9 | 1174.7 | 173.8 | 0.857 |
| Optic tract | 34.4 | 19.2 | 31.8 | 19.4 | 0.603 |
| Lenticular fasciclus | 448.3 | 99.3 | 442.9 | 74.4 | 0.815 |
| Nucleus accumbens | 2057.3 | 192.9 | 2014.5 | 188.5 | 0.401 |
| Nucleus innominata of Mynert | 851 | 173.7 | 841.6 | 122.1 | 0.814 |
| Hypothalamus | 564 | 72.5 | 573 | 71.5 | 0.641 |
| LVL_frontal | 4367.4 | 2248.7 | 4968.1 | 2539 | 0.351 |
| LVL_body | 3355.4 | 1708.7 | 3605.2 | 1653.3 | 0.577 |
| LVL_atrium | 2234.6 | 1150.7 | 2570.7 | 1280.7 | 0.304 |
| LVL_occiptal | 165.8 | 102 | 186.5 | 143.9 | 0.537 |
| LVL_temporal | 1594 | 498.5 | 1684.4 | 534.3 | 0.513 |
| IV ventricle | 8375.6 | 1513.2 | 8809.2 | 1484.3 | 0.280 |
| SLF-t | 2626.6 | 426.7 | 2593.7 | 575.2 | 0.809 |
| İntra cerebellum | 610 | 109.8 | 623.6 | 95.2 | 0.617 |
| cerebellum branch A | 3685.5 | 800.9 | 3669.3 | 655 | 0.933 |
| cerebellum branch B | 1060 | 226.7 | 1004.6 | 300.4 | 0.439 |
| cerebro-spinal fluid | 453536.5 | 32195 | 455915.6 | 31190.5 | 0.778 |

**Supplementary Material (Appendices)**

**Appendix B**

**Table 5.** Baseline characteristics of left cerebral hemisphere measurements

|  | **Group** | | | | **p** |
| --- | --- | --- | --- | --- | --- |
|  | **Healthy (n=31)** | | **Patient (n=32)** | |  |
|  | **Mean** | **SD** | **Mean** | **SD** |  |
| Superior parietal gyrus | 26717.6 | 3145.5 | 26354.0 | 2420.1 | 0.630 |
| Cingulate gyrus | 30029.4 | 2295.8 | 30529.9 | 1855.0 | 0.373 |
| Superior frontal gyrus | 53529.0 | 3087.8 | 53442.3 | 2815.7 | 0.913 |
| Middle frontal gyrus | 53327.4 | 3802.5 | 53237.2 | 3874.9 | 0.930 |
| İnferior frontal gyrus | 29079.1 | 3125.2 | 28433.7 | 2494.3 | 0.396 |
| Precentral gyrus | 28962.7 | 2203.3 | 28420.9 | 1981.3 | 0.335 |
| Postcentral gyrus | 29313.6 | 1706.6 | 29964.2 | 1892.1 | 0.178 |
| Angular gyrus | 13396.5 | 1341.8 | 13239.7 | 1367.6 | 0.664 |
| Pre-cuneus | 14382.3 | 1382.5 | 14481.8 | 1360.9 | 0.786 |
| Cuneus | 14733.4 | 2082.5 | 15024.5 | 2035.3 | 0.596 |
| Lingual gyrus | 20093.4 | 2132.0 | 20856.0 | 2538.6 | 0.223 |
| Fusiform gyrus | 30983.4 | 2146.1 | 30804.4 | 1700.5 | 0.730 |
| Parahippocampal gyrus | 1578.8 | 246.4 | 1338.1 | 123.9 | **< .001** |
| Superior occipital gyrus | 5809.6 | 765.9 | 5586.8 | 967.7 | 0.337 |
| Inferior occipital gyrus | 6914.2 | 1010.1 | 6867.6 | 1034.9 | 0.864 |
| Middle occipital gyrus | 32197.5 | 3620.0 | 31735.5 | 2713.8 | 0.591 |
| Entorhinal area | 1504.0 | 390.4 | 1391.6 | 237.5 | 0.202 |
| Superior temporal gyrus | 42679.6 | 2391.3 | 42541.3 | 3319.8 | 0.857 |
| Inferior temporal gyrus | 19275.1 | 1521.4 | 19115.3 | 1799.6 | 0.718 |
| Middle temporal gyrus | 43706.4 | 2673.8 | 42841.5 | 3069.8 | 0.261 |
| Lateral fronto-orbital gyrus | 14205.2 | 1278.6 | 13825.8 | 1250.6 | 0.263 |
| Middle fronto-orbital gyrus | 8839.6 | 760.4 | 8751.5 | 933.7 | 0.697 |
| Supramarginal gyrus | 22097.4 | 2057.5 | 22097.3 | 1468.1 | 1.000 |
| Gyrus rectus | 10588.9 | 1223.0 | 10491.7 | 1287.4 | 0.771 |
| Insular | 9492.4 | 754.5 | 9323.5 | 791.3 | 0.413 |
| Amygdala | 2213.6 | 539.6 | 2221.2 | 502.7 | 0.956 |
| Hippocampus | 6443.4 | 571.6 | 6254.6 | 522.4 | 0.200 |
| Cerebellum | 93170.3 | 7748.1 | 95474.0 | 6975.6 | 0.245 |
| Corticospinal tract | 1039.5 | 302.1 | 953.8 | 303.7 | 0.291 |
| Inferior cerebellar peduncle | 1057.3 | 209.4 | 971.9 | 178.4 | 0.105 |
| Medial lemniscus | 1535.2 | 230.3 | 1480.0 | 412.1 | 0.531 |
| Superior cerebellar peduncle | 1777.5 | 227.6 | 1723.5 | 222.7 | 0.371 |
| Cerebral peduncle | 2005.9 | 280.0 | 1953.5 | 253.7 | 0.464 |
| Anterior limb of internal capsule | 1988.1 | 368.2 | 2028.9 | 259.7 | 0.634 |
| Posterior limb of internal capsule | 4179.0 | 463.2 | 4294.4 | 480.8 | 0.361 |
| Posterior thalamic radiation | 14461.3 | 1651.0 | 14091.0 | 1391.0 | 0.367 |
| Anterior corona radiata | 9208.2 | 1527.8 | 9448.9 | 1389.1 | 0.538 |
| Superior corona radiata | 12778.2 | 1514.4 | 12443.3 | 1513.2 | 0.408 |
| Posterior corona radiata | 6929.6 | 863.9 | 6860.0 | 863.3 | 0.763 |
| Cingulum (cingulate gyrus) | 2942.8 | 649.8 | 2914.9 | 727.4 | 0.879 |
| Cingulum (hippocampus) | 1546.7 | 304.5 | 1581.6 | 334.8 | 0.682 |
| Fornix (cres) / Stria terminalis left | 1046.4 | 169.2 | 1088.4 | 183.5 | 0.373 |
| Superior longitudinal fasciculus | 9830.2 | 1101.6 | 9323.7 | 1338.9 | 0.123 |
| Inferior fronto-occipital fasciculus | 2824.9 | 391.4 | 2783.9 | 319.6 | 0.669 |
| Sagittal stratum | 7060.1 | 839.4 | 7319.0 | 733.4 | 0.223 |
| External capsule | 3738.7 | 358.0 | 3815.7 | 484.0 | 0.495 |
| Uncinate fasciculus | 1598.6 | 253.4 | 1605.1 | 252.7 | 0.922 |
| Pontine crossing tract | 1378.8 | 411.7 | 1574.2 | 389.3 | 0.072 |
| Middle cerebellar peduncle | 8521.0 | 1182.0 | 8772.6 | 1140.6 | 0.418 |
| Fornix (column and body of fornix) | 269.9 | 76.4 | 265.4 | 86.3 | 0.836 |
| Genu of corpus callosum | 3177.9 | 551.0 | 3106.8 | 432.4 | 0.593 |
| Body of corpus callosum | 4692.4 | 461.7 | 4463.0 | 586.9 | 0.105 |
| Splenium of corpus callosum | 7911.7 | 669.6 | 6746.6 | 770.1 | **< .001** |
| Retrolenticular part of internal capsule | 2226.4 | 266.8 | 2243.4 | 291.9 | 0.819 |
| Red Nucleus | 53.2 | 35.8 | 55.7 | 28.9 | 0.773 |
| Substancia nigra | 141.6 | 76.1 | 142.0 | 59.3 | 0.982 |
| Tapatum | 1408.4 | 241.8 | 1095.4 | 151.9 | **< .001** |
| Caudate nucleus | 5325.2 | 533.2 | 5382.9 | 600.9 | 0.703 |
| Putamen | 4477.6 | 492.2 | 4397.8 | 388.3 | 0.503 |
| Thalamus | 9413.7 | 557.6 | 9138.1 | 680.3 | 0.099 |
| Globus pallidus | 1095.9 | 168.6 | 1083.1 | 155.8 | 0.768 |
| Midbrain | 2826.4 | 260.9 | 2896.4 | 291.0 | 0.342 |
| Pons | 369.9 | 109.4 | 325.1 | 140.0 | 0.182 |
| Medulla | 3127.4 | 442.2 | 3065.4 | 416.9 | 0.589 |
| Anterior commissure | 154.4 | 57.7 | 151.5 | 36.9 | 0.824 |
| Ansa lenticularis | 1307.3 | 161.8 | 1262.0 | 169.4 | 0.307 |
| Optic tract | 43.0 | 40.8 | 62.2 | 51.0 | 0.122 |
| Lenticular fasciclus | 520.9 | 134.8 | 497.6 | 95.3 | 0.459 |
| Nucleus accumbens | 1971.6 | 183.7 | 1760.3 | 121.0 | **< .001** |
| Nucleus innominata of Mynert | 597.9 | 138.4 | 623.8 | 115.8 | 0.450 |
| Hypothalamus | 616.2 | 97.6 | 610.7 | 79.6 | 0.816 |
| LVL_frontal | 5676.8 | 2108.0 | 6188.0 | 2379.7 | 0.394 |
| LVL_body | 4148.9 | 1883.2 | 4430.1 | 1927.1 | 0.580 |
| LVL_atrium | 2238.7 | 1502.5 | 2585.5 | 1278.0 | 0.355 |
| LVL_occiptal | 355.6 | 288.5 | 458.6 | 356.7 | 0.234 |
| LVL_temporal | 1052.5 | 497.8 | 1209.5 | 370.1 | 0.187 |
| IV ventricle | 8375.6 | 1513.2 | 8809.2 | 1484.3 | 0.280 |
| SLF-t | 4281.7 | 709.9 | 4274.0 | 746.2 | 0.968 |
| İntra cerebellum | 459.3 | 94.9 | 468.1 | 121.7 | 0.762 |
| Cerebellum branch A | 3970.4 | 843.8 | 3941.2 | 658.4 | 0.886 |
| Cerebellum branch B | 1046.1 | 307.6 | 912.1 | 351.2 | 0.130 |
| Cerebro-spinal fluid | 453536.5 | 32195.0 | 455915.6 | 31190.5 | 0.778 |

**Supplementary Material (Appendices)**

**Appendix C**

| **Table 6.** Performance metrics (mean and 95% confidence interval) of algorithms on repeated cross-validation of training set for left cerebral hemisphere measurements | | | | |
| --- | --- | --- | --- | --- |
| **Model/Metric** | **Accuracy** | **AUC** | **Brier** | **F1-Score** |
| Logistic Regression | 0.4533  (0.3838, 0.5229) | 0.4725  (0.3945, 0.5505) | 0.5442  (0.4751, 0.6133) | 0.4915  (0.4099, 0.5730) |
| KNN | 0.5700  (0.4953, 0.6447) | 0.6900  (0.5999, 0.7801) | 0.2501  (0.2081, 0.2921) | 0.6090  (0.5301, 0.6880) |
| Svm (Polynomial) | 0.7350  (0.6783, 0.7917) | 0.7950  (0.7212, 0.8688) | 0.2035  (0.1783, 0.2286) | 0.7340  (0.6688, 0.7992) |
| Svm (Radial) | 0.7000  (0.6303, 0.7697) | 0.7750  (0.6951, 0.8549) | 0.2140  (0.1855, 0.2425) | 0.7489  (0.6856, 0.8123) |
| Random Forest | 0.8367  (0.7830.0.8903) | 0.8950  (0.8433, 0.9467) | 0.1720  (0.1583, 0.1856) | 0.8604  (0.8102, 0.9106) |
| LDA | 0.6883  (0.6212, 0.7555) | 0.7600  (0.6708, 0.8492) | 0.2655  (0.2051, 0.3258) | 0.7514  (0.6938, 0.8091) |
| MLP | 0.6867  (0.6155, 0.7579) | 0.7550  (0.6764, 0.8336) | 0.2156  (0.1842, 0.2470) | 0.7090  (0.6388, 0.7793) |
| C5 | 0.6667  (0.6021, 0.7313) | 0.6750  (0.6080.0.7420) | 0.3299  (0.2663, 0.3935) | 0.6752  (0.6008, 0.7496) |
| XGBoost | 0.8317  (0.7823, 0.8810) | 0.9025  (0.8452, 0.9598) | 0.1132  (0.0864, 0.1400) | 0.8483  (0.8021, 0.8945) |
| MARS | 0.7183  (0.6469, 0.7897) | 0.7675  (0.6919, 0.8431) | 0.2778  (0.2076, 0.3480) | 0.7048  (0.6191, 0.7904) |
| **AUC:** area under the receiver operating curve.  *Naive Bayes and Bagging algorithms were excluded due to the fact that the corresponding results could not be obtained in left cerebral hemisphere measurements. | | | | |

| **Table 7.** Performance metrics (mean and 95% confidence interval) of algorithms performance based on independent testing set for left cerebral hemisphere measurements | | | | |
| --- | --- | --- | --- | --- |
| **Model/Metric** | **Accuracy** | **AUC** | **Brier** | **F1-Score** |
| Logistic Regression | **0.6300**  (0.5361, 0.7239) | 0.5444  (0.4180.0.6709) | 0.3701  (0.2762, 0.4641) | **0.7197**  (0.6205, 0.8189) |
| KNN | 0.5500  (0.4460.0.6540) | 0.5778  (0.4323, 0.7233) | 0.2482  (0.2042, 0.2922) | 0.6500  (0.5284, 0.7716) |
| Svm (Polynomial) | 0.5700  (0.4593, 0.6807) | 0.4222  (0.2733, 0.5711) | 0.2867  (0.2493, 0.3242) | 0.6752  (0.5468, 0.8036) |
| Svm (Radial) | 0.4900  (0.4449, 0.5351) | 0.4444  (0.2946, 0.5943) | 0.2718  (0.2544, 0.2893) | 0.6809  (0.6610.0.7007) |
| Random Forest | 0.5700  (0.4710.0.6690) | 0.6444  (0.5001, 0.7888) | **0.2378**  (0.2266, 0.2491) | 0.7009  (0.5919, 0.8098) |
| LDA | 0.5900  (0.4843, 0.6957) | **0.7556**  (0.6260.0.8851) | 0.2545  (0.2105, 0.2986) | 0.7037  (0.6016, 0.8058) |
| MLP | 0.5800  (0.4756, 0.6844) | 0.6333  (0.5008, 0.7659) | 0.2666  (0.2198, 0.3133) | 0.6842  (0.5586, 0.8098) |
| C5 | 0.4600  (0.3463, 0.5737) | 0.5111  (0.3944, 0.6278) | 0.5325  (0.4203, 0.6447) | 0.5455  (0.3844, 0.7065) |
| XGBoost | 0.5100  (0.4175, 0.6025) | 0.3778  (0.2316, 0.5240) | 0.3142  (0.2787, 0.3498) | 0.5985  (0.4910.0.7059) |
| MARS | 0.5500  (0.4679, 0.6321) | 0.5222  (0.3953, 0.6492) | 0.4368  (0.3582, 0.5153) | 0.6556  (0.5219, 0.7892) |
| AUC: area under the receiver operating curve.  *Naive Bayes and Bagging algorithms were excluded due to the fact that the corresponding results could not be obtained in left cerebral hemisphere measurements. | | | | |
